# Forecasting framework for dominant SARS-CoV-2 strains before clade replacement using phylogeny-informed genetic distances

**DOI:** 10.3389/fmicb.2025.1619546

**Published:** 2025-06-20

**Authors:** Kyuyoung Lee, Atanas V. Demirev, Sangyi Lee, Seunghye Cho, Hyunbeen Kim, Junhyung Cho, Jeong-Sun Yang, Kyung-Chang Kim, Joo-Yeon Lee, Woojin Shin, Soyoung Lee, Sejik Park, Philippe Lemey, Man-Seong Park, Jin Il Kim

**Affiliations:** ^1^Department of Microbiology, Institute for Viral Diseases, Korea University College of Medicine, Seoul, Republic of Korea; ^2^Division of Emerging Viral Diseases and Vector Research, Center for Infectious Diseases Research, National Institute of Infectious Diseases, Korea National Institute of Health, Osong, Republic of Korea; ^3^Center for Infectious Diseases Research, National Institute of Infectious Diseases, Korea National Institute of Health, Osong, Republic of Korea; ^4^Department of Microbiology, Immunology, and Transplantation, Rega Institute, KU Leuven, Leuven, Belgium; ^5^Vaccine Innovation Center, Korea University College of Medicine, Seoul, Republic of Korea; ^6^Biosafety Center, Korea University College of Medicine, Seoul, Republic of Korea

**Keywords:** SARS-CoV-2, evolution, clade replacement, forecasting framework, spike gene, dominance

## Abstract

**Introduction:**

Severe acute respiratory syndrome coronavirus 2 (SARS-CoV-2) is the causative agent of the global coronavirus disease 2019 (COVID-19) pandemic and continues to drive successive waves of infection through the emergence of novel variants. Consequently, accurately predicting the next clade roots through global surveillance is crucial for effective prevention, control, and timely updates of vaccine antigen updates. This study evaluated the evolutionary dynamics of SARS-CoV-2 using phylogeny-informed genetic distances based on 394 complete genomes and spike (S) gene sequences. Furthermore, we introduced a forecasting framework to estimate the potential of emerging variants leading to clade replacement by analyzing non-synonymous and synonymous genetic distances from clade roots, which reflect global herd immune pressure.

**Methods:**

Non-synonymous and synonymous genetic distances from both Wuhan and clade root strains were assessed to predict whether a clade would become dominant or extinct within 3 months before the clade replacement.

**Results:**

Through five observed clade replacements up to January 2024, we captured the quantifiable heterogeneity in non-synonymous and synonymous genetic distances of the S gene from clade roots between dominant and extinct variants, as measured by the extent of novelty, whether through gradual or drastic change.

**Discussion:**

Our framework demonstrated high predictability for identifying the next clade root before replacement in both training and test datasets (area under the receiver operating characteristic curve [AUROC] > 0.90) by incorporating differential weighting of non-synonymous and synonymous genetic distances. Additionally, the framework solely using spike gene data demonstrated similar accuracy to those using the complete genome. Overall, our approach establishes quantifiable molecular criteria for identifying potential updates to the SARS-CoV-2 vaccine, contributing to proactive pandemic preparedness.

## 1 Introduction

Severe acute respiratory syndrome coronavirus 2 (SARS-CoV-2) is the viral pathogen responsible for the global coronavirus disease 2019 (COVID-19) pandemic (Zhu et al., 2020). Since its emergence in late 2019, various SARS-CoV-2 variants have emerged, cycling through phases of spread and extinction. Some variants, notably Delta and Omicron, have effectively displaced earlier strains, leading to subsequent waves of infection. These dominant variants exhibited amino acid mutations, particularly in the spike (S) protein, which is a crucial mediator of host cell entry and a primary target of the adaptive immune response (Obermeyer et al., 2022). Such mutations likely enhance viral fitness by improving transmissibility, replication efficiency, and evasion of adaptive immune responses (Dadonaite et al., 2024; Maher et al., 2022; Markov et al., 2023; Starr et al., 2022). As a result, the ongoing emergence of variants with significant mutations in the S protein complicated the antibody-driven prevention and treatment efforts against COVID-19.

The global surveillance of SARS-CoV-2 novel variants is essential for effective prevention and control, particularly in identifying clade replacements that may indicate substantial shifts in transmissibility or antigenicity, prompting updates to vaccine strategies (Demirev et al., 2023; Morris et al., 2018). Neutralization assays demonstrate the antibody-driven immune response against a variant, similar to the hemagglutination inhibition (HI) test used in seasonal influenza virus surveillance (Huddleston and Bedford, 2024; Smith et al., 2004). The cross-reactivity measured by neutralization assays can be mapped to the antigenicity of SARS-CoV-2 variants, indicating their fitness under hosts’ immune pressure (Koel et al., 2013; Planas et al., 2024; Wilks et al., 2023). The change in the antigenicity of variants informs the determination of updates to s or therapeutics, considering their potential for transmissibility through breakthrough infections (Willett et al., 2022). However, SARS-CoV-2 neutralization assays are still being improved for experimental standardization. Furthermore, similar to the HI test, SARS-CoV-2 neutralization assays encounter delays in confirming the antigenicity of the many globally reported variants in real time, as they require moderate time and resources.

Computational approaches, supported by global data-sharing platforms of genomic surveillance, help identify high-potential dominant variants (Gardy and Loman, 2018; Matteson et al., 2023; Shu and McCauley, 2017). The World Health Organization (WHO) coordinates the monitoring and classification of variants, designating specific “variants of concern” (VOC) based on significant mutations in the S gene (SG) (WHO, 2021). Although the VOC classification scheme provides a detailed framework, its meticulous criteria may hinder the timely identification of variants with high potential for clade replacement. Artificial intelligence (AI)-driven protein structure models also provide insights into variant fitness by predicting biochemical characteristics and interactions between surface proteins and antibodies (Abramson et al., 2024; Krishna et al., 2024). However, this approach remains limited by computational and methodological constraints related to the limited availability of viral protein data. Phylogenetic methods are robust tools for elucidating viral evolutionary dynamics, especially for RNA viruses with high mutation rates (Grenfell et al., 2004; Holmes and Grenfell, 2009). Multiple tree-based methods have been employed to detect emerging RNA virus variants and identify dominant variants likely to cause clade replacements (Huddleston et al., 2020; Łuksza and Lässig, 2014; Neher et al., 2014). Despite the strength of phylogeny-informed approaches, genetic distance metrics derived from immune-driven strains have been sparingly to identify replacements within SARS-CoV-2 clades (Kistler et al., 2022; Morris et al., 2018; Perofsky et al., 2024).

Our study evaluated phylogeny-informed genetic distances from key immune-driven VOCs shaping global herd immunity using complete genome (CG) and SG sequences as statistical predictors of clade replacement from the onset of the COVID-19 pandemic to January 2024. Additionally, we introduced a forecasting framework to quantify the potential impact of emerging variants likely to lead to the upcoming clade replacement.

## 2 Materials and methods

### 2.1 Data collection

The present study collected 394 CG sequences and their epidemiological information of global SARS-CoV-2 clade-definable strains subsampled by the Nextstrain team, the globally-renowned SARS-CoV-2 molecular epidemiology research group (Hadfield et al., 2018), from the EpiCoV database of the Global Initiative on Sharing All Influenza Data (GISAID)^1^ (Shu and McCauley, 2017), reported from December 2019 to January 2024 (Supplementary Data S1). The sequences were aligned with Multiple Alignment using Fast Fourier Transform (MAFFT) (v7.419, RIMD, Japan) (Katoh and Standley, 2013) and finalized under manual review. Stop codons were removed from each ORF, and one cysteine nucleotide was inserted at nucleotide position 13,203 to ensure the continuity of the open reading frame of the ORF1a and ORF1b genes and the three-letter nature of codons. The alignments of gene segments were concatenated following the order of ORF1a,b (1–21,288), spike (21,289–25,116), ORF3 (25,117–25,941), E gene (25,941–26,166), M gene (26,167–26,832), ORF6 (26,834–27,015), ORF7 (27,016–27,378), ORF8 (27,379–27,741), N gene (27,742–28,998), and ORF10 (28,999–29,112) from 5′ end to 3′ end using SeaView (v4, PRABI. France) (Gouy et al., 2010) (Supplementary Data S2). The alignment of spike gene (SG) sequences was extracted from the CG sequences (Supplementary Data S3). We also collected 143 SG sequences and their epidemiological information of global SARS-CoV-2 clade-definable strains subsampled by the Nextstrain team from the EpiCoV database of the GISAID reported from January to October 2024 for the cross-validation of our forecasting framework. We combined the original (*n* = 394) and additional (*n* = 143) SG sequences, performing the same steps of cleaning and alignment to use as test data for cross-validation in the forecasting framework (*n* = 537) (Supplementary Data S4).

### 2.2 Bayesian phylogeny and tree-informed genetic distances estimation

CG and SG alignments were used for the phylogeny estimation by Bayesian Markov chain Monte Carlo (MCMC) Metropolis–Hastings algorithm on Bayesian evolutionary analysis sampling tree (BEAST) (v1.10.4. BEAST Developers) (Suchard et al., 2018). We used the generalized time-reversible substitution model, uncorrelated lognormal relaxed molecular clock, Bayesian renaissance counting (RNSC) (Lemey et al., 2012), and Bayesian skygrid prior model (Hill and Baele, 2019) with 12 parameters (2 parameters per year) and 6 years at the last transition point (the year of SARS-CoV-2 emergence: 2019). The MCMC chain length was initiated with 300 million runs, followed by a 10% burn-in period, and increased until the MCMC chains reached reliable convergence and stationarity. The convergence and stationarity of MCMC chains for numerical estimates were assessed using the effective sample size (ESS) and tracer plot in Tracer (v1.7.2. BEAST Developers). The estimation was accepted if the ESSs of all continuous estimates were higher than 100 with well-mixed tracer plots. The maximum clade credibility (MCC) trees of CG and SG were summarized from tree samples of MCMC estimation using TreeAnnotator (v.1..10.5, BEAST Developers) and visualized by the “ggtree” package (v1.14.6, Bioconductor) (Yu et al., 2017) on R Studio (v.4.4.0, R Studio, Inc. MA, US).

Synonymous and non-synonymous genetic distances from a clade root (CR) strain numerically explain the extent of genetic heterogeneity in a variant compared to immune-driven strains, and it was measured by counting substitutions from a CR strain in a phylogeny. The present study measured two types of genetic distances separately. First, the genetic distances from the Wuhan strain demonstrate the extent of genetic heterogeneity caused by the immune pressure influenced by the origin of the SARS-CoV-2 strain. Second, the genetic distances from the CR strain illustrate the extent of genetic heterogeneity arising from the immune pressure formed after clade replacement. For the Bayesian estimation of genetic distances, a total of 500 phylogenies of CG and SG were randomly selected from the tree logs of the MCMC estimation. We selected the CR strains that became the origin of the dominant variant clade after clade replacement. The synonymous and non-synonymous distances from the CR strain and Wuhan strain were extracted as phylogeny-informed genetic distances of tips from the 500 sampled phylogenies of CG and SG using an in-house script on R Studio. The 500 synonymous and nonsynonymous genetic distances for each strain were summarized as the median and 95% upper and lower boundaries to investigate statistical variability in a point estimate. The Pearson correlation coefficients were estimated to evaluate the statistical association between synonymous and non-synonymous genetic distances of CG and SG.

### 2.3 Antigenic distance estimation in Bayesian cartography

The present study collected the metadata of SARS-CoV-2 neutralization titers from Wuhan to JN.1 strains through two research publications by Wilks et al. (2023) and Planas et al. (2024) (Supplementary Data S5). A total of 5,689 neutralization titers, along with their collection year, were used to estimate the first- and second-dimension coordinates using Bayesian estimation of antigenic cartography (Bedford et al., 2014). The MCMC chain length was initiated with 1 billion runs, followed by a 10% burn-in period, and then increased until the MCMC chains reached reliable convergence and stationarity. The convergence and stationarity of MCMC chains for numeric estimates were assessed using ESS and a tracer plot in Tracer. The estimation was accepted if the ESSs of all continuous estimate projections were higher than 200 with well-mixed tracer plots. The clade of 394 SARS-CoV-2 strains was classified using nucleotide sequences of the SG with Wuhan-Hu-1 with XBB SNPs as reference (updated: 17 October 20120) by the Nextclade (Aksamentov et al., 2021) (Supplementary Data S1). A total of 106 strains (106/394, 26.9%) were successfully matched with the clades in the base antigenic cartography (Supplementary Figure S1). The point estimates of the first- and second-dimension coordinates of 106 strains and 5 serums were summarized in the median under the investigation of value distribution. The Euclidean distance between strains and serum of the CR and Wuhan strains in the antigenic cartography was evaluated as the antigenic distance. The Pearson correlation coefficients were estimated to assess the statistical association of antigenic distance with synonymous and non-synonymous genetic distances of CG and SG.

### 2.4 Statistics in forecasting framework to quantify the potential of the next dominant strains

The present study targeted SARS-CoV-2 strains that were spread 3 months before the clade replacement, considering these as the viral population with maximum genetic diversity. The strains were labeled either “Dominant” if a strain was involved in the clade that diverged from the Most Recent Common Ancestor (MRCA) of the next CR strains in the MCC phylogenies of CG and SG, or “Extinct.” The Wilcoxon signed-rank test was used to assess the statistical association between dominance and synonymous and nonsynonymous genetic distances, as measured by CG and SG, considering the violation of the normality assumption due to the low sample size (*n* < 30) in each CR group. The optimal cut-off value of genetic distances for the classification of highly novel dominant variants was determined by the threshold in the receiver operating characteristic (ROC) curve analysis. A multivariable logistic regression model was fitted to evaluate the statistical association between dominance and synonymous and non-synonymous genetic distances from Wuhan or CR of CG and SG.


$$y_{i}\sim \text{Binomial}\;(n_{i},\pi_{j})$$

$$\text{logit}\;(\pi_{i})=\beta_{o}+\beta_{\text{Non}-\mathrm{syn}}X_{\text{Non}-\mathrm{syn}\;i}+\beta_{\mathrm{Syn}}X_{\mathrm{Syn}\;i}$$

where:

$y_{i}\;$ is the dominance of a strain *i* (0 = Extinct/1 = Dominant).

$\pi_{i}\;$ is the expected probability of dominance of a strain *i*.

$X_{\text{Non}-\mathrm{syn}},X_{\mathrm{Syn}}$ is the non-synonymous and synonymous genetic distances from the CR strain.

$\beta_{o}$ is the base intercept.

$\beta_{\text{Nonsyn}},\beta_{\mathrm{Syn}}$ is the log odds ratio (weight) for predictor variables.

The backward model selection was performed to identify statistically significant independent variables among synonymous and non-synonymous genetic distances in the final regression model by comparing the Akaike information criterion (AIC) and the Wald test. The regression analysis and visualization were performed on R Studio. Statistical significance was determined using a *p*-value threshold of 0.05. The generalizability of the model’s predictability was evaluated through cross-validation of the area under the receiver operating characteristic curve (AUROC) between the training and test data. Model 1 included *α*-*β*-*γ* and Delta CR groups as test data and performed cross-validation using test data from BA.2 and XBB.1.5 CR groups. Model 2 involved α-β-γ to XBB.1.5 CR groups as test data. Model 2 is used to evaluate the coherence of statistical estimates with Model 1 and to perform the prediction of the next CR strain from the JN.1. strain in October 2024 as indicated in the second data.

## 3 Results

### 3.1 The emergence of global SARS-CoV-2 variants and clade replacements

**Figure 1:**
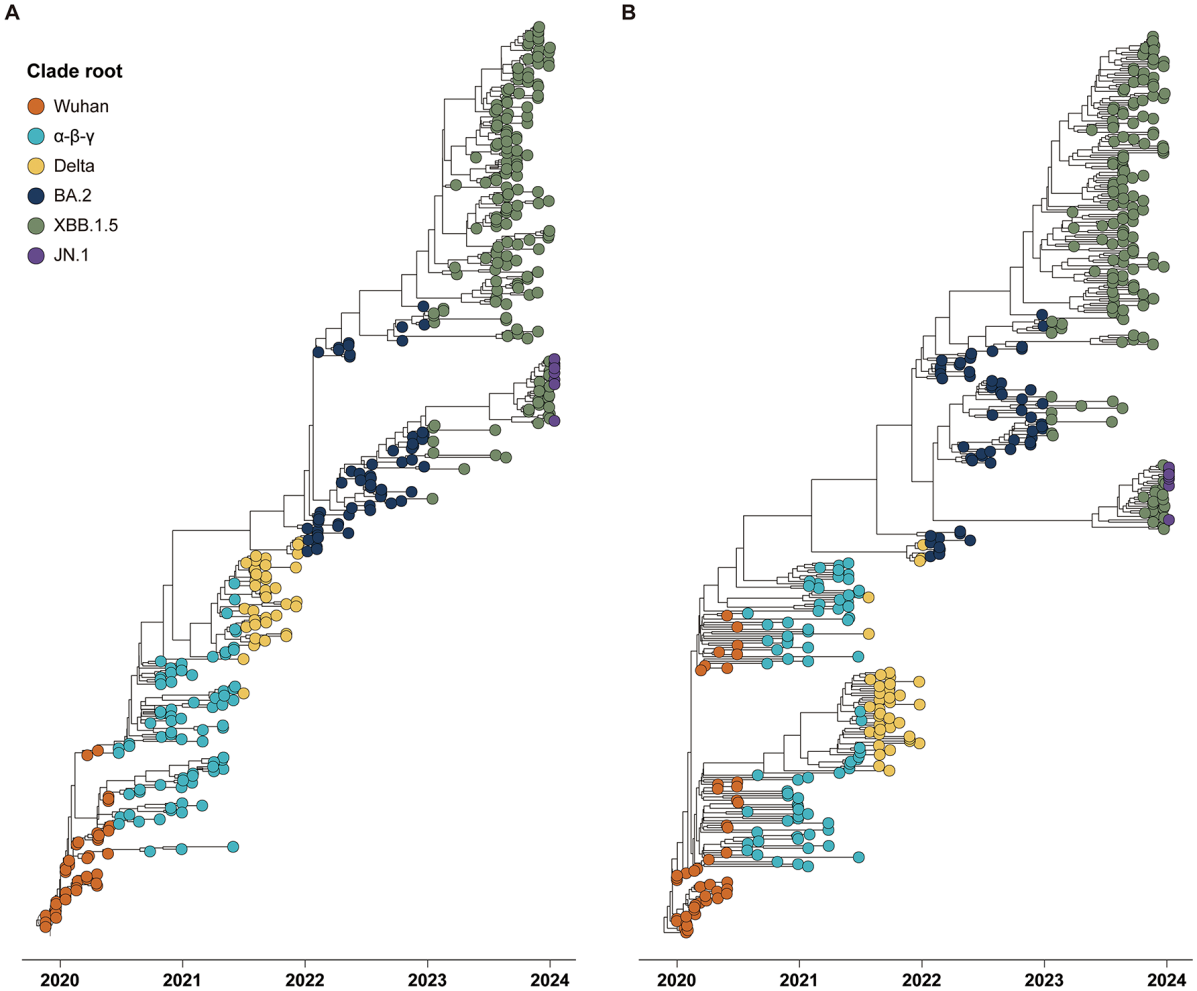
Phylogenies of 394 global SARS-CoV-2 strains reported from December 2019 to January 2024 with tips colored by the period of six clade roots. **(A)** Time phylogeny estimated by spike gene sequences. **(B)** Time phylogeny estimated by complete genome sequences.

The phylogenies of CG and SG depicted five major clade replacements through a ladder-shaped topology comprised of the rapid growth of a novel clade and subsequent local extinction of other clades (Figure 1). The first two clade replacements, from the Wuhan to the α-β-γ clades, and from the α-β-γ to the Delta clades, were observed around July 2020 and July 2021, respectively. Following the emergence of the Delta clade, the three subsequent clade replacements were observed at the end of 2022, 2023, and 2024. The Wuhan and Delta clades dominated for approximately 6 months, respectively, and each of the other three clades (α-β-γ, BA.2, and XBB.1.5) dominated for almost 1 year until the subsequent clade replacements.

### 3.2 Estimation of genetic distances from Wuhan or clade root strains in the phylogenies

Following the period of the five clade replacements, we classified 394 representative SARS-CoV-2 strains into six clade root (CR) groups (Table 1). The extent of genetic heterogeneity in a variant against immune-driven strains was numerically measured by counting synonymous and non-synonymous substitutions from a CR strain in phylogenies of CG and SG, referred to as genetic distances. We separately measured two types of genetic distances. First, the genetic distances from the Wuhan strain illustrate the extent of gene heterogeneity resulting from the immune pressure shaped by the origin of the SARS-CoV-2 strain. Second, the genetic distances from the CR strain explain the extent of gene heterogeneity resulting from the immune pressure formed after clade replacement.

**Table 1:** The number of SARS-CoV-2 strains classified by clade root (CR) after the clade replacement.

Clade root	Period	Number of strains
Wuhan	December 2019–to June 2020	47
α-β-γ	June 2020–to June 2021	79
Delta	July 2021–December 2021	40
BA.2	January 2022–December 2022	56
XBB.1.5	January 2023–December 2023	164
JN.1	January 2024	8
	Total	394

**Figure 2:**
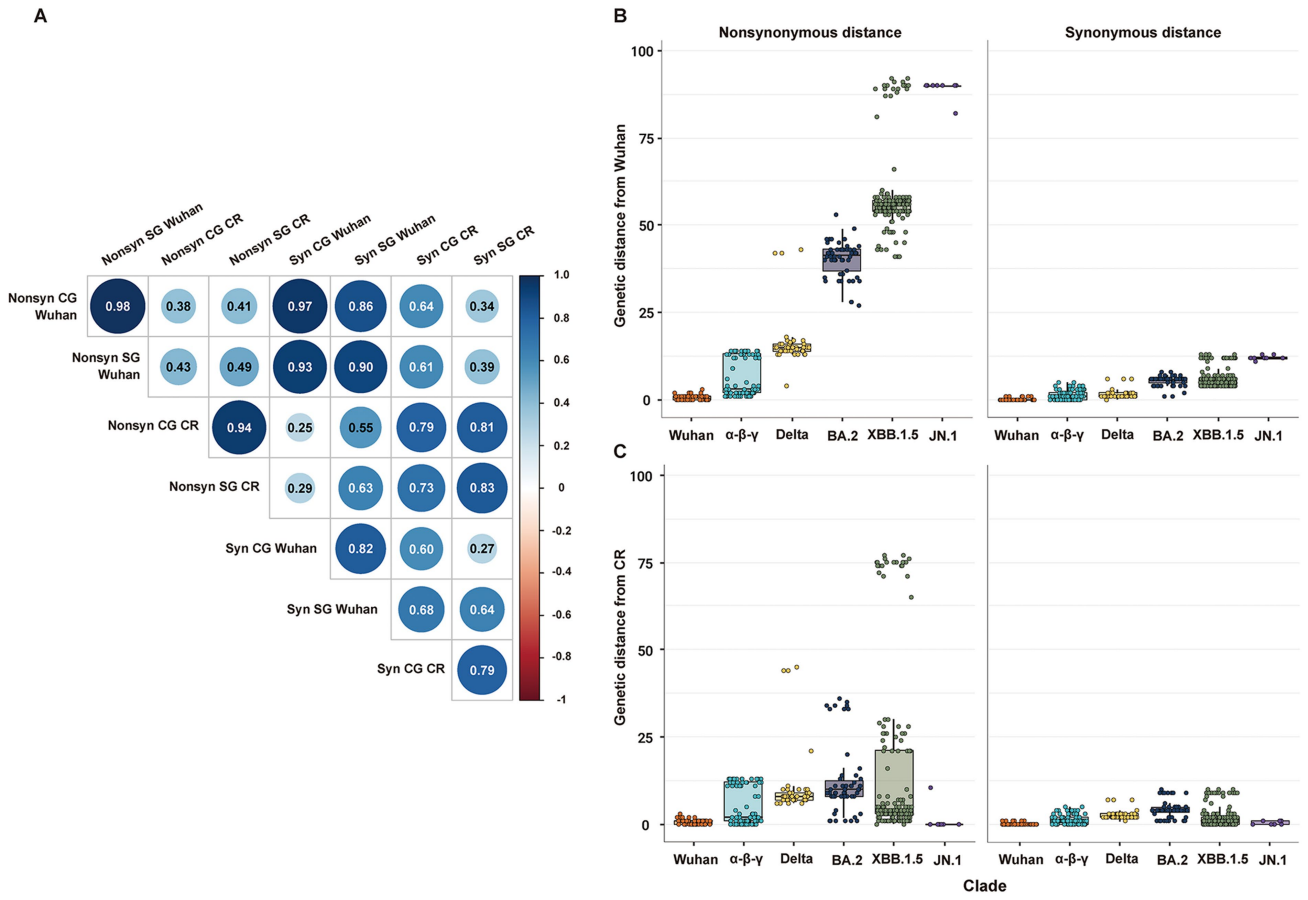
Distribution of non-synonymous and synonymous genetic distances of SG or CG from Wuhan or CR. **(A)** Correlation matrix of non-synonymous and synonymous genetic distances of SG or CG from Wuhan or CR. **(B)** Distribution of non-synonymous and synonymous genetic distances of SG from Wuhan. **(C)** Distribution of non-synonymous and synonymous genetic distances of SG from CR.

Genetic distances estimated from CG and SG phylogenies showed a statistically significant positive correlation. Specifically, non-synonymous and synonymous genetic distances had a very high positive correlation between each pair of CG and SG (Figure 2A). The genetic distances from the Wuhan strain measured the temporal increment of all mutations since the first emergence of SARS-CoV-2 (Figure 2B). On the other hand, the genetic distances from the CR strain measured the temporal increment of mutations partially during the period of each CR group (Figure 2C). Every non-synonymous genetic distance had higher variability than its synonymous genetic distances in both CG and SG (Figure 2; Supplementary Figure S2).

The Bayesian statistical variability in a point estimate of the genetic distances was higher in CG than in SG (Supplementary Figure S3). Specifically, a few strains exhibited relatively high statistical variability in the estimation of non-synonymous genetic distance of CG due to incongruence at local branches, which classified recombinant strains such as BA.2 and JN.1 (Supplementary Figures S3a–c).

### 3.3 The association between phylogeny-informed genetic distances and antigenic distances

**Figure 3:**
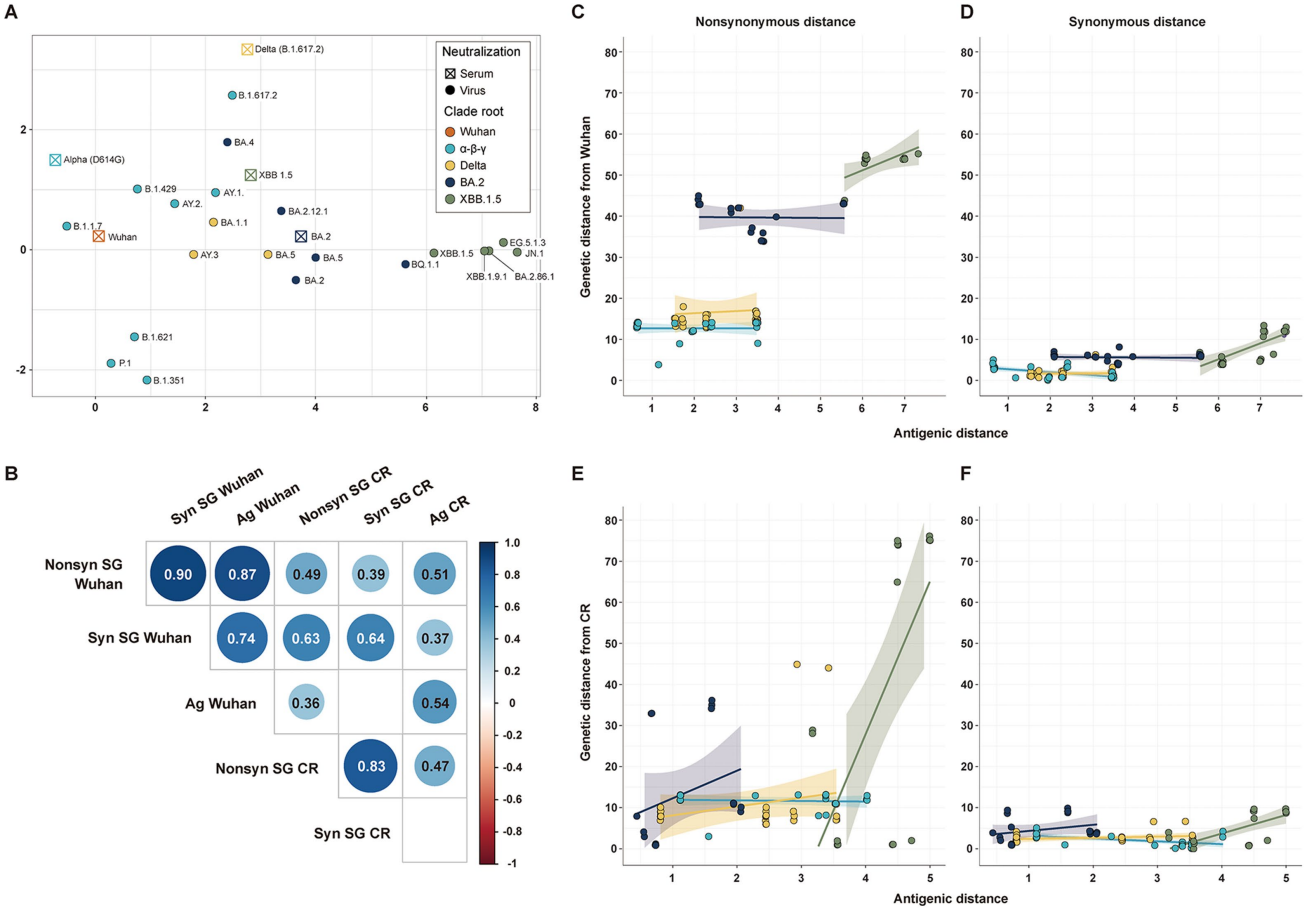
Correlation among antigenic and genetic distances of SG. **(A)** Antigenic cartography with 5 serums and 21 strains. **(B)** Correlation matrix among the four genetic distances of SG and the antigenic distance. **(C)** Correlation between the non-synonymous genetic distance of SG and antigenic distance from Wuhan. **(D)** Correlation between the synonymous genetic distance of SG and antigenic distance from Wuhan. **(E)** Correlation between the non-synonymous genetic distance of SG and antigenic distance from CR. **(F)** Correlation between the synonymous genetic distance of SG and the antigenic distance from CR.

The antigenic cartography was used to estimate the antigenic distance of SARS-CoV-2 strains from Wuhan (*n* = 106) and CR antisera (*n* = 101) (Figure 3A). All genetic distances of SG showed a statistically significant positive correlation with antigenic distances, except the synonymous genetic distance from the CR strain. Non-synonymous genetic distances had higher correlation coefficients with antigenic distances than synonymous genetic distances (Figure 3B). The genetic distance of SG from the Wuhan strain showed a high correlation coefficient with antigenic distance, due to a clear difference in antigenicity among CR groups. Despite the lower correlation coefficients, genetic distances of SG from the CR strain distinctly displayed an association with the antigenic distance among variants in the same CR group rather than those from Wuhan (Figures 3C–F). The Delta, BA.2, and XBB1.5 CR groups had a positive correlation in both non-synonymous and synonymous genetic distances with antigenic distances (Figures 3E,F). On the other hand, variants in the *α*-*β*-*γ* CR group exhibited a negative correlation between synonymous genetic distance and antigenic distance (Figures 3D,F).

All genetic distances of CG exhibited a statistically significant positive correlation with antigenic distances, similar to those of SG. Even the synonymous genetic distances from the CR showed a significant positive correlation with antigenic distances (Supplementary Figure S4).

### 3.4 The heterogeneity in genetic distances between dominant and extinct strains before the clade replacement

**Figure 4:**
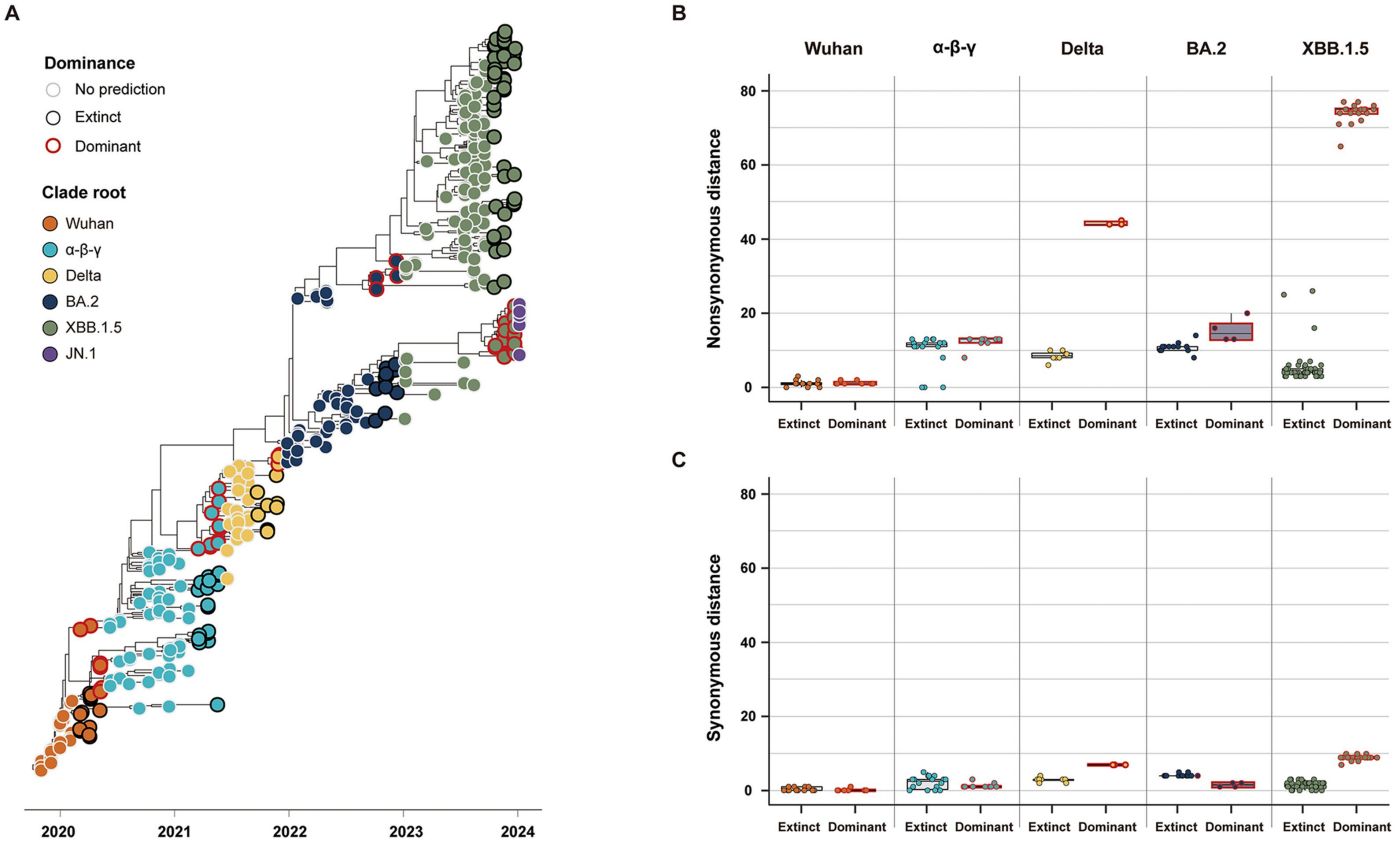
Classification and genetic distance distribution between dominant and extinct strains. **(A)** Phylogenies of SG classifying dominant and extinct strains among variants reported 3 months before the clade replacement. **(B)** Distribution of non-synonymous genetic distance from CR in SG between dominant and extinct strains. **(C)** Distribution of synonymous genetic distance from CR in SG between dominant and extinct strains.

Our study classified the 141 variants reported 3 months before the five clade replacements into either dominant or extinct strains, based on the phylogenies of SG (Figure 4A). In the five CR groups, excluding the JN.1 group, a total of 46 strains were labeled as the dominant strain, and the other 95 strains were labeled as the extinct strain (Dominant: Extinct ≈ 1:2) (Table 2). The dominant strains commonly had higher non-synonymous genetic distances from the CR strain in SG than the extinct strains (Figure 4B; Supplementary Table S1). However, in the α-β-γ and BA.2 CR groups, the dominant strain had lower or the synonymous genetic distances than the extinct strain. In contrast, the dominant strains in the Delta and XBB.1.5 CR groups had much higher synonymous genetic distances than the extinct strains (Figure 4C). The Wuhan CR group did not show a significant difference between dominant and extinct strains in both genetic distances from the CR strain (Supplementary Table S1).

**Table 2:** The number of dominant and extinct strains in the prediction models using genetic distances from CR in SG.

Clade root	Periods	Number of extinct strains	Number of dominant strains	Total Number of strains	Model 1	Model 2
Wuhan	April 2020–June 2020	12	8	20		–
α-β-γ	April 2021–June 2021	18	10	28	Train	Train
Delta	October 2021–December 2021	8	3	11	Train	Train
BA.2	October 2022–December 2022	12	4	16	Test	Train
XBB.1.5	October 2023–December 2023	45	21	66	Test	Train
JN.1	October 2024			44	–	Test
	Total	95	46	174		

The phylogeny of CG was also used to classify either dominant or extinct strains (Supplementary Figure S5a). Dominant and extinct strains exhibited high coherence in the distribution of genetic distances in CG, similar to those in SG (Supplementary Figures S5b,c; Supplementary Table S1). However, due to incongruent topology in phylogenies between CG and SG, around 8.7% of dominant strains in SG (4/46) were classified as extinct strains in CG, and 14.3% of dominant strains in CG (7/49) were classified as extinct strains in SG (Supplementary Table S2).

### 3.5 The forecasting framework of the next CR strain before the clade replacement using genetic distances

**Figure 5:**
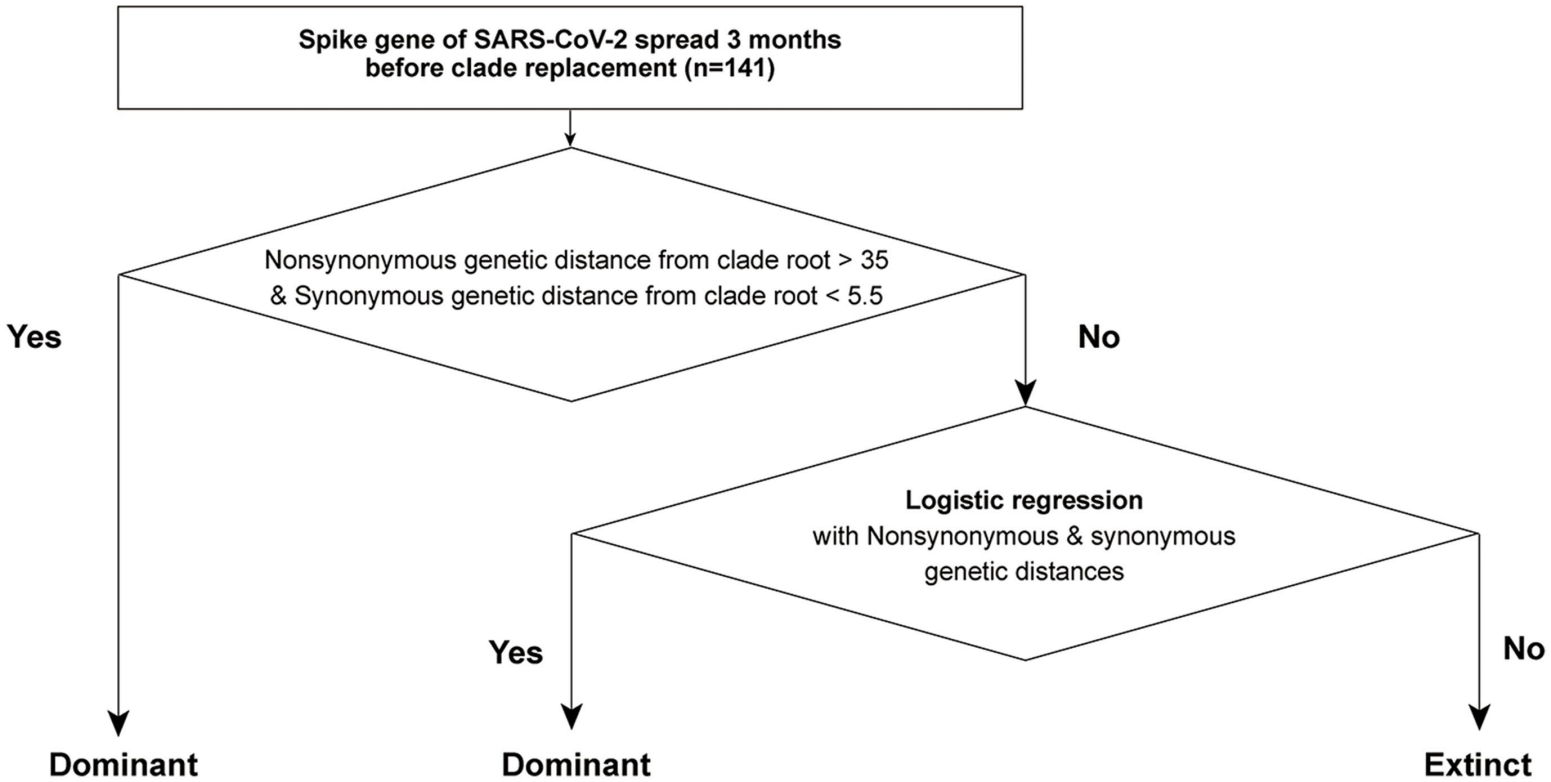
The scheme of a two-step forecasting framework using genetic distances from CR in SG.

Considering the heterogeneity of non-synonymous and synonymous genetic distances between dominant and extinct strains, we established a two-step forecasting framework using genetic distances from the CR strain in SG (Figure 5). The first step detected “genetically highly novel” variants, and this dominant strain exhibited high non-synonymous and synonymous genetic distances from the CR strain (e.g., the BA.2 strains from the Delta CR group and JN.1 strains from the XBB.1.5 CR group). The optimal cut-off values for classifying the dominant strain were estimated to be over 35 for non-synonymous and 5.5 for synonymous genetic distances from the CR strain in SG. In the second step, all strains not selected as the dominant strain in the first step were tested, and multivariable logistic regression was fitted with different weights based on either non-synonymous or synonymous genetic distances from the CR strain.

The present study evaluated the predictability of the model through cross-validation, where the training and test data were subsets by the CR group. In Model 1, the strains in the *α*-*β*-*γ* and Delta CR groups were used as training data, and those in BA.2 and XBB.1.5 CR groups were used as test data (Table 2). The estimates from the second step in Model 1 using SG showed a positive association between non-synonymous genetic distance and a negative association between synonymous genetic distance and the dominance of variants (Table 3). The odds ratio indicated that the count of a non-synonymous mutation increased the likelihood of dominance by approximately 2.6 times. In contrast, the accumulation of synonymous mutations decreases the possibility of dominance to about 0.3 times. Model 1 in SG exhibited high predictability, with an area under the receiver operating characteristic curve (AUROC) of over 0.90 in both training (0.925) and testing (=0.963) datasets (Table 4; Supplementary Figure S6).

**Table 3:** Statistical estimates of the second step in the forecasting frameworks with the multivariable logistic regression using SG and CG.

Data	Model	Variable	Estimate	Odds ratio	*p*-value
SG	1	Non-synonymous distance	0.937	2.55 (0.94, 6.91)	0.065
		Synonymous distance	−1.080	0.34 (0.14, 0.82)	0.016
	2	Non-synonymous distance	1.113	3.04 (1.17, 7.95)	0.023
		Synonymous distance	−1.292	0.27 (0.13, 0.62)	0.002
CG	1	Non-synonymous distance	0.257	1.29 (1.05, 1.60)	0.018
		Synonymous distance	−0.957	0.38 (0.13, 1.12)	0.080
	2	Non-synonymous distance	0.138	1.15 (1.03, 1.28)	0.013
		Synonymous distance	−0.415	0.66 (0.51, 0.85)	0.002

**Table 4:** The area under the curve of the receiver operating characteristics (AUROC) in the forecasting framework of models 1 and 2 was estimated using SG and CG.

Gene	Model	Dataset for cross-validation	AUROC
SG	1	Train data	0.925
SG	1	Test data	0.963
SG	2	Train data	0.963
CG	1	Train data	0.975
CG	1	Test data	0.958
CG	2	Train data	0.955

We also fitted a forecasting framework of CG with a similar scheme to the model in SG (Supplementary Figure S7). The optimal cut-off values for the classification of the first step, detecting “genetically highly novel” dominant strain, were estimated to be over 68.5 for non-synonymous and 22.5 for synonymous genetic distances from the CR strain in CG (Supplementary Figure S7). The second step also involved fitting multivariable logistic regression with different weights based on non-synonymous or synonymous genetic distances from CR for all variant strains that were not selected as the dominant strain in the first step. The second step of Model 1 in CG showed the same direction of association between dominance and genetic distances, but had around half the estimate of non-synonymous genetic distance compared to that in SG. However, it showed high similarity in the forecast of synonymous genetic distance (Table 3). Model 1 in CG also exhibited high predictability, with an AUROC of over 0.95 in both training (0.975) and test data (0.958) (Table 4; Supplementary Figure S6).

### 3.6 The forecasting framework of emerging variants in the upcoming clade replacement using genetic distances of SG

**Figure 6:**
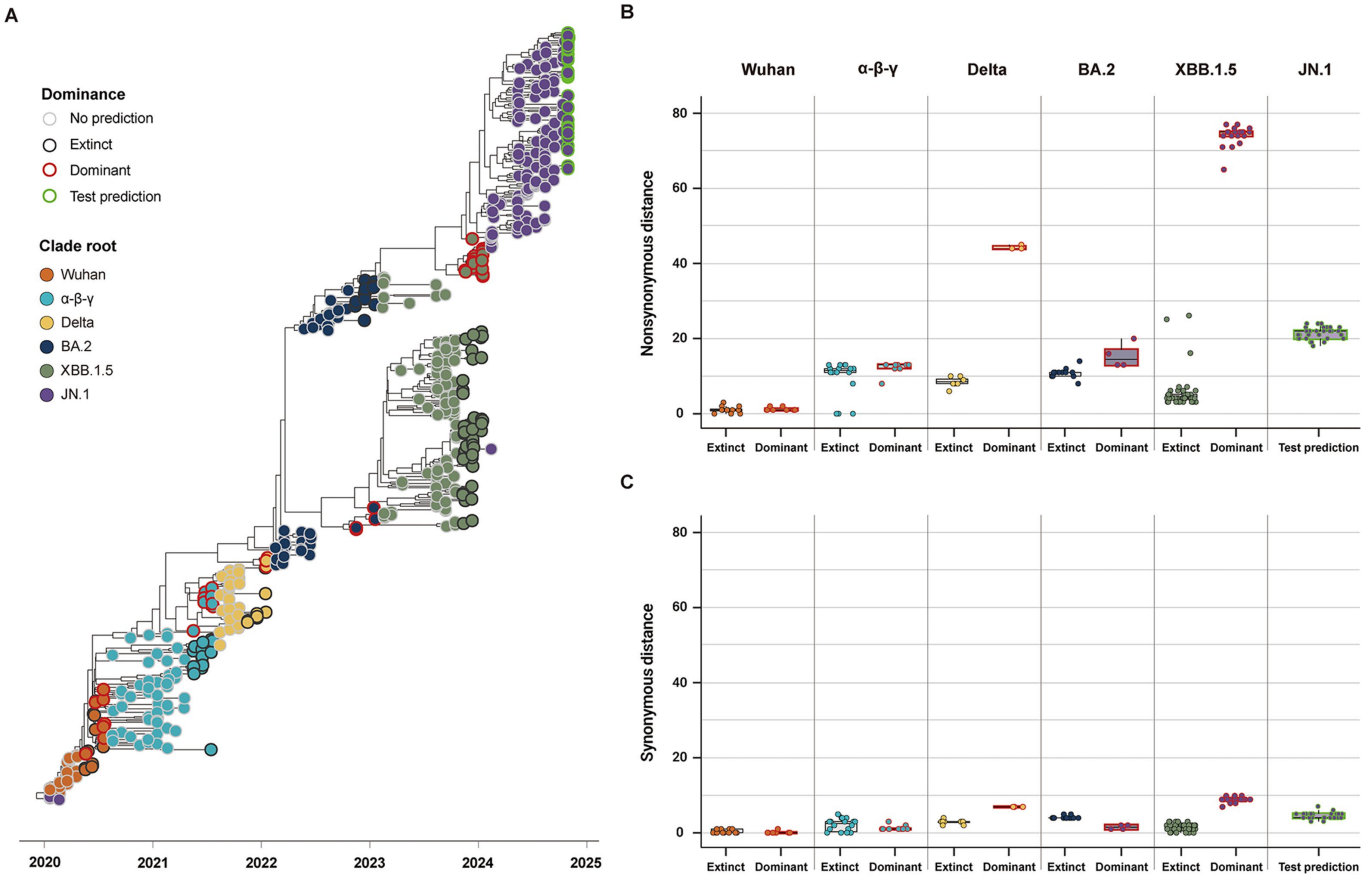
Classification and genetic distance distribution between dominant and extinct strains in the second data for the cross-validation collected until October 2024 (*n* = 537). **(A)** Phylogenies of SG classifying dominant and extinct strains among variants reported 3 months before the clade replacement. **(B)** Distribution of non-synonymous genetic distance from CR in SG between dominant and extinct strains. **(C)** Distribution of synonymous genetic distance from CR in SG between dominant and extinct strains.

Considering the high comparability of the forecasting framework between SG and CG, we used only SG in the additional 146 SARS-CoV-2 strains reported as of October 2024 for the second cross-validation. The phylogeny of SG with the second cross-validation data revealed that 145 strains originated from the JN.1 lineage, and one strain from Russia in September was classified as an XBB strain (Figure 6A). In Model 2, the strains from *α*-*β*-*γ* to XBB.1.5 CR groups were involved as the training data, and 44 strains in the JN.1 CR group were involved as the test data (Table 2). The 44 strains in the test data were from 18 to 24 and from 3 to 7 in non-synonymous and synonymous genetic distances from the CR, respectively (Figures 6B,C). They did not strictly meet the criteria in the first step of the forecasting framework for high genetic novelty. The second step of Model 2 in SG showed high numeric coherence in estimates, as well as the direction of association between dominance and genetic distances (Table 3). The model 2 in SG also exhibited high predictability, with an AUROC in the training data (= 0.963) and similar to as that in CG (= 0.955) (Table 4; Supplementary Figure 6). The second step of Model 2 yielded a high dominance score (> 0.999) for many strains of the test data, despite being emerging variants at an early stage, due to their very high non-synonymous genetic distances compared to the early form of the JN.1 strain reported in November 2023 (Table 5). Particularly, the KP.3.1.1. and LB.1.3.1 sublineages reported in October 2024 had a relatively high dominance score among the 44 JN.1 strains.

**Table 5:** The dominance score of the top seven strains and their non-synonymous and synonymous genetic distances to SG from CR.

Rank	Accession number	Country	Sublineage	Prediction score	Non-synonymous genetic distance from JN.1.	Synonymous genetic distance from JN.1
1	PQ461534	USA	MC.2	>0.9999	24	4
1	PQ536516	USA	KP.3.1.1	>0.9999	24	4
3	PQ525133	USA	XDY	>0.9999	24	5
4	PQ461550	USA	KP.3.1.1	>0.9999	23	4
4	PQ509644	USA	LB.1.3.1	>0.9999	23	4
4	OZ198117	Denmark	KP.3.1.1	>0.9999	23	4
4	PQ509566	USA	LB.1.3.1	>0.9999	23	4

## 4 Discussion

The present study investigated the evolutionary dynamics of SARS-CoV-2, specifically focusing on the genetic heterogeneity among variants reported before the clade replacements. Using phylogeny-informed genetic distances with differential weighting based on the selection pressure, we effectively quantified the viral fitness of variants. We estimated the potential of emerging variants that would likely lead to upcoming clade replacement.

The phylogenies of both the CG and SG coherently depicted the five distinct clade replacements through the evolution of global SARS-CoV-2 variants from the Wuhan to JN.1 clades. The phylogeny of the CG and SG showed strong temporal structure comprising a high rate of local clade extinction and continual replacement of successful clades through the strong viral fitness difference, as other rapidly evolving RNA viruses showed (e.g., Influenza virus) (Grenfell et al., 2004). The epidemic of SARS-CoV-2 led to an increasing number of infected people, as well as expanding the genetic pools by the emergence of novel variants. While the genetic pool of SARS-CoV-2 shrank after the peak of the epidemic, the population of variants is forced to naturally select the next CR due to the difference in viral fitness. Antigenicity, shaped by immune pressure, is a significant evolutionary feature influencing viral fitness. SG, a pivotal genetic component of antigenicity, appeared to reliably represent the evolutionary patterns of SARS-CoV-2 associated with clade replacement, demonstrating strong concordance with CG (Kistler et al., 2022).

Our study investigated quantifiable evolutionary features in SG, differentiating between the dominant and extinct strains before clade replacement. The first clade replacement from the Wuhan to *α*-*β*-*γ* CR group did not show clear numeric metrics. Antigenic clusters, as well as genetic distances, did not show a clear, quantifiable difference between dominant and extinct strains in the Wuhan CR group. However, the SG of the dominant strain in the Wuhan CR group exhibited low genetic heterogeneity, which is inconsistent with that of the CG. This possibly demonstrates that the clade replacement of the Wuhan group was driven by the global fixation of the D614G mutation in SG under extensive purifying selection in other genomic components, which likely optimized host adaptation by promoting efficient human-to-human transmission during the early stages of the pandemic (Antia et al., 2003; Korber et al., 2020; Rochman et al., 2021). From the second clade replacement within the α-β-γ CR group, we could measure metrics of evolutionary features in SG that differentiate between the dominant and extinct strains. After the emergence of the D614G variant, including the α-β-γ CR group, the mutation in key residues of SG for efficient antibody evasion determined viral fitness by clades and drove the emergence of regional variants (Rochman et al., 2021).

The present study characterized two genetic criteria of the next CR strain that will lead to clade replacement. The first type of dominant strain was genetically highly novel, numerically characterized by high non-synonymous and synonymous genetic distances of the SG as well as the CG. The replacement of BA.2 from the Delta clade, and JN.1 from the XBB.1.5 clade, possibly exemplified the first type of genetic characteristics of the dominant strain. These dominant strains exhibited dramatically high antigenic novelty compared to concurrent strains, resulting from the genetic recombination of key gene regions that express antigenicity, particularly the N-terminal and receptor binding domains in the SG (Demirev et al., 2023). The first step of our model is designed to capture the dramatic genetic novelty of the dominant strain by high non-synonymous and synonymous genetic distances from the CR strain in the SG.

The replacement of Delta strains from the α-β-γ clade, and XBB.1.5 strains from the BA.2 clade, likely exhibited the second type of genetic characteristics of the dominant strain, which emerged through antigenic drift. The second type of dominant strain is numerically defined by the accumulation of amino acid mutations in key antigenic residues, while maintaining minimal mutations in conserved residues in both SG and CG. The second step of our model is designed to classify either dominant or extinct strains based on non-synonymous and synonymous genetic distances from the CR strain with differential weighting through a multivariable logistic regression model. Models 1 and 2 explained that a variant with an amino acid mutation of SG showed 2–3 times higher likelihood of dominance than others, but a variant with a synonymous mutation had 3 times lower likelihood. A high non-synonymous genetic distance from the CR strain can numerically illustrate the gradual natural selection of the dominant strain (Łuksza and Lässig, 2014). Like the seasonal emergence of novel influenza A strains, SARS-CoV-2 has promoted the accumulation of advantageous mutations that evade the host’s adaptive immunity, which is developed through prior infections or vaccinations. On the other hand, a low synonymous genetic distance in the dominant strain possibly implies a small number of deleterious mutations in genetically conserved residues under purifying selection (Koelle and Rasmussen, 2015).

Our study designs a two-step forecast framework to detect the next CR after the clade placement using phylogeny-informed genetic distances of SG with differential weighting. A phylogeny straightforwardly depicts the hierarchy of viral evolution, specifically on nucleotide level. However, phylogenetic topology and branch length only partially illustrate viral fitness due to the heterogeneity of selection pressure on the amino acid residue (Demirev et al., 2023; Harvey et al., 2016; Koel et al., 2013; Koelle and Rasmussen, 2015; Zhang et al., 2022). The present study separately measured non-synonymous and synonymous genetic distances from a phylogeny, assigned separate statistical weights to quantify their differential effects on immune-driven selection pressure and viral fitness, leading to clade replacement, and showed high predictability (AUROC > 0.9). However, model 2 achieved high scores in multiple sublineages and did not identify a dominant strain among the 44 strains in the test data. Interestingly, the 2024–2025 period did not show a clear clade replacement by one variant and multiple sublineages such as KP.3.1.1, XEC, LP.8.1, LF.7, and NB.1.8.1 coexisted with similar composition of genetic diversity among geographical regions until May 2025 (Hadfield et al., 2018). Our forecasting framework also seemed to capture comparably high viral fitness of multiple variants, which has made it difficult for any single variant to lead the clade replacement.

Our study tested two criteria of our numeric predictors, genetic distances, considering (1) the comparability of SG with CG and (2) the different impact of immune pressure shaped by either the origin of the SARS-CoV-2 strain, or the CR strain formed after clade replacement. Then our model targeted the global SARS-CoV-2 strains reported 3 months before the clade replacement, focusing on maximum genetic pools prior to the clade replacement, which commonly occurred from October to December in the Northern Hemisphere.

The genetic distances of the SG seemed to sufficiently explain evolutionary characteristics related to the clade replacement and antigenic distance as much as those of CG. The phylogeny of SG may not fully capture the evolutionary attributes of SARS-CoV-2 driven by other genetic components in CG (Wagner et al., 2024). However, our study found that the genetic distances of the SG were highly correlated with antigenic distances, and the predictability of the models was also highly comparable to that of CG. This seems reasonable because most evolutionary processes driving clade replacement, such as genetic recombination and antigenic drift, are commonly observed in the SG rather than in another genetic region (Dadonaite et al., 2024; Harvey et al., 2021; Jackson et al., 2021; Mittal et al., 2022). Furthermore, considering the low cost of computational resources and high stability of point estimates in Bayesian estimation, genetic distances of SG could be more preferable predictors than those of CG to capture the viral fitness related to the clade replacement.

The genetic distances from the CR strain properly quantified the viral fitness related to immune pressure before the clade replacement. The “spindly” phylogeny of SARS-CoV-2 illustrates the phylodynamics with rapid clade replacement through a short infectious period and the host population’s partial cross-immunity (Ferguson et al., 2003; Grenfell et al., 2004; Koelle et al., 2006). Even with vaccine- or infection-induced immunity in the global population, primarily driven by the Wuhan strain during the pandemic, the global population’s immune pressure appeared to be rapidly reshaped by serial clade replacements and/or booster vaccine administration (Huang et al., 2023). Our antigenic cartography also illustrated that the cluster of novel CR strain was located around the cluster of prior CR strains. Therefore, genetic distances from CR effectively quantify the viral fitness of a variant, conditioned on the serial shifts in host immune pressure caused by clade replacement.

Despite our meaningful findings, our results may not capture the full phylogenetic history of SARS-CoV-2, including every clade-specific key mutation possibly related to viral fitness. We estimated the pruned phylogenies using the genetic sequences of clade-definable strains and a model based on the clade-definable trees, which likely excluded clade-specific evolutionary history and overestimated the predictability. However, our phylogenies would reflect major evolutionary characteristics, including key mutations significantly related to viral fitness, and suggest informative numeric predictors to detect strong candidates that could lead to a clade replacement. Our next step will refine the forecasting framework by using the genetic characteristics of other genetic domains (e.g., Nsp6 and ORF7a) (Kistler et al., 2022) and incorporating more clade-specific evolutionary history of extinct strains to capture key residues related to purifying selection (Neher, 2022).

Even so, we would address the limited generalizability of the seasonal pattern of clade placement. Over the last 3 years, SARS-CoV-2 has shown a regularity in the pattern of clade replacement, repeating the expansion of genetic diversity through an increasing number of infection cases and natural selection of a strong candidate by either antigenic drift or the emergence of highly novel variants. Our study appears to reveal generalizable evolutionary characteristics that may have led to the dominant candidate, possibly contributing to clade replacement through extrapolation of evolutionary characteristics of RNA viruses with high seasonality, such as the influenza virus. However, we still pondered whether six years of the global SARS-CoV-2 pandemic would be sufficient to determine whether an evolutionary pattern is an outcome of contingency or a key deterministic driver of natural selection with high repeatability (Beavan et al., 2024; Blount et al., 2018). Furthermore, our framework considered antigenic response to host immune pressure as the sole determinant of viral fitness, under the assumption of a seasonally homogeneous transition of herd immunity driven by clade replacement. During the pandemic, the global population simultaneously acquired herd immunity to SARS-CoV-2 variants through infection and/or vaccination. However, in the post-pandemic era, immune imprinting is likely to contribute to heterogeneity in herd immunity across birth cohorts, vaccination histories, and the geographical distribution of emerging variants, potentially influencing the evolutionary dynamics of SARS-CoV-2 (Barrat-Charlaix et al., 2021; Bedford et al., 2015; Chemaitelly et al., 2023; Gostic et al., 2016; Huang et al., 2023; Koutsakos and Ellebedy, 2023; Lemey et al., 2020; Russell et al., 2008; Tegally et al., 2023; Tortorici et al., 2024; Vieira et al., 2021). Therefore, our next step will be to expand the forecasting framework to incorporate epidemiological and immunological characteristics, aiming for better predictability in the post-pandemic era.

## 5 Conclusion

Optimal selection for the update of vaccine strain against rapidly evolving viruses plays an essential role in endorsing high vaccine effectiveness (Huddleston and Bedford, 2024; McAdams, 2014). Our study focused on the repeating pattern of clade replacement by the emergence of genetically novel variants of SARS-CoV-2, specifically following a 3-month window of seasonal outbreaks in the Northern Hemisphere. Furthermore, the present study revealed that phylogeny-informed non-synonymous and synonymous genetic distances of SG from CR appear to be plausible predictors for inferring the future CR strain before clade replacement, considering the sampling scale, the time and resources required for analysis, the convenience of information size, and predictability. We believe that our intuitive, simple, but potent forecasting framework could provide better insight for the current global SARS-CoV-2 prevention and control measures under the technical advantage of future genomic surveillance (Morris et al., 2018).

## Data Availability

The datasets generated and analyzed for this study can be found in the Supplementary materials.

## Footnotes

^1^ http://www.gisaid.org
