# Supplementary material for: Forecasting framework for dominant SARS-CoV-2 strains before clade replacement using phylogeny-informed genetic distances
Source: Front Microbiol. 2025 Jun 20;16:1619546. doi: 10.3389/fmicb.2025.1619546 (PMC12226564; doi:10.3389/fmicb.2025.1619546)
Supplement: Supplementary file 2 [file Data_Sheet_2.zip › S1 table.docx]

**SUPPLEMENTAL TABLE**

**Supplementary Table 1. The p-values of Wilcoxon rank sum test for the statistical difference of nonsynonymous and synonymous genetic distances of SG and CG from CR between dominant and extinct strains 3 months before the clade replacement.**

| Clade root | Genetic data | Nonsynonymous | Synonymous |
| --- | --- | --- | --- |
| Wuhan | SG | 0.511 | 0.329 |
|  | CG | 0.789 | 0.225 |
| α-β-γ | SG | 0.016 | 0.276 |
|  | CG | 0.004 | 0.007 |
| Delta | SG | 0.017 | 0.012 |
|  | CG | 0.018 | 0.017 |
| BA.2 | SG | 0.007 | 0.001 |
|  | CG | 0.20 | 0.017 |
| XBB.1.5 | SG | < 0.001 | < 0.001 |
|  | CG | < 0.001 | < 0.001 |
