# Supplementary material for: Forecasting framework for dominant SARS-CoV-2 strains before clade replacement using phylogeny-informed genetic distances
Source: Front Microbiol. 2025 Jun 20;16:1619546. doi: 10.3389/fmicb.2025.1619546 (PMC12226564; doi:10.3389/fmicb.2025.1619546)
Supplement: Supplementary file 2 [file Data_Sheet_2.zip › S2 table.docx]

**SUPPLEMENTAL TABLE**

**Supplementary Table 2. The number of dominant and extinct strains in the two-step forecasting frameworks before the clade replacement using genetic distances of complete genome from clade root.**

| Clade root | Periods | Number of extinct strains | Number of dominant strains | Total Number of strains | Model 1 | Model 2 |
| --- | --- | --- | --- | --- | --- | --- |
| Wuhan | 2020, April - 2020, June | 5 | 15 | 20 |  | - |
| α-β-γ | 2021, April - 2021, June | 19 | 9 | 28 | Train | Train |
| Delta | 2021, Oct. - 2021, Dec. | 8 | 3 | 11 | Train | Train |
| BA.2 | 2022, Oct. - 2022, Dec. | 12 | 4 | 16 | Test | Train |
| XBB.1.5 | 2023, Oct. - 2023, Dec. | 48 | 18 | 66 | Test | Train |
|  | Total | 92 | 49 | 141 |  |  |
