# Supplementary material for: Forecasting framework for dominant SARS-CoV-2 strains before clade replacement using phylogeny-informed genetic distances
Source: Front Microbiol. 2025 Jun 20;16:1619546. doi: 10.3389/fmicb.2025.1619546 (PMC12226564; doi:10.3389/fmicb.2025.1619546)
Supplement: Supplementary file 2 [file Data_Sheet_2.zip › Supplementary_Material_Captions.pdf]

frontiers

Supplementary Material

1 Supplementary Data

Supplementary Data 1. Metadata of SARS-CoV-2 strain sequence globally collected from Dec. 2019 to Oct. 2024 (n = 537)

Supplementary Data 2. Concatenated complete genome sequence alignment of SARS-CoV-2 strains globally collected from Dec. 2019 to Jan. 2024 (n = 394)

Supplementary Data 3. Spike gene sequence alignment of SARS-CoV-2 strains globally collected from Dec. 2019 to Jan. 2024 (n = 394)

Supplementary Data 4. Spike gene sequence alignment of SARS-CoV-2 strains globally collected from Dec. 2019 to Oct. 2024 used as the test data of Model 2 (n = 537)

Supplementary Data 5. Metadata of 5,689 SARS-CoV-2 neutralization titer for the Bayesian antigenic cartography estimation between 20 serum and 42 strains

2 Supplementary Figures and Tables

2.1 Supplementary Figures

Supplementary Figure 2. The antigenic cartography of SARS-CoV-2 between 20 serum and 42 strains. The present study performed the Bayesian antigenic cartography using the metadata of neutralization titer (n = 5,689)

Supplementary Figure 2. Distribution of nonsynonymous and synonymous genetic distances of CG from Wuhan or CR. (A) Distribution of nonsynonymous and synonymous genetic distances of CG from Wuhan. (B) Distribution of nonsynonymous and synonymous genetic distances of CG from CR.

Supplementary Figure 3. Statistical variability of a point estimate through Bayesian estimation. (A) Nonsynonymous genetic distances of CG from Wuhan strain. (B) Synonymous genetic distances of CG from Wuhan strain. (C) Nonsynonymous genetic distances of CG from CR strain. (D) Synonymous genetic distances of CG from CR strain. (E) Nonsynonymous genetic distances of SG from Wuhan strain. (F) Synonymous genetic distances of SG from Wuhan strain. (G) Nonsynonymous genetic distances of CG from CR strain. (H) Synonymous genetic distances of CG from CR strain.

Supplementary Figure 4. Correlation among antigenic and genetic distances of CG. (A) Correlation matrix among four genetic distances of CG and antigenic distance. (B) Correlation between nonsynonymous genetic distance of CG and antigenic distance from Wuhan. (C) Correlation between synonymous genetic distance of CG and antigenic distance from Wuhan. (D) Correlation between

nonsynonymous genetic distance of CG and antigenic distance from CR. €. Correlation between synonymous genetic distance of CG and antigenic distance from CR.

Supplementary Figure 5. Classification and genetic distance distribution of CG between dominant and extinct strains. (A) Phylogenies of CG classifying dominant and extinct strains among variants reported 3 months before the clade replacement. (B) Distribution of nonsynonymous genetic distance from CR in CG between dominant and extinct strains. (C) Distribution of synonymous genetic distance from CR in CG between dominant and extinct strains.

Supplementary Figure 6. The evaluation of predictability for two-step forecasting framework by cross-validation. The ROC curves of (A) Train, (B) Test data of model 1 and (C) Test data of model 2 using the phylogeny of the SG, (D) Train, (E) Test data of model 1 and (F) Test data of model 2 using the phylogeny of the CG.

Supplementary Figure 7. The scheme of two-step forecasting framework using genetic distances from CR in the CG.

2.2 Supplementary Table

Supplementary Table 1. The p-values of Wilcoxon rank sum test for the statistical difference of nonsynonymous and synonymous genetic distances of SG and CG from CR between dominant and extinct strains 3 months before the clade replacement.

Supplementary Table 2. The number of dominant and extinct strains in the two-step forecasting frameworks before the clade replacement using genetic distances of complete genome from clade root.
