# Supplementary figures and images for: Forecasting framework for dominant SARS-CoV-2 strains before clade replacement using phylogeny-informed genetic distances
Source: Front Microbiol. 2025 Jun 20;16:1619546. doi: 10.3389/fmicb.2025.1619546 (PMC12226564; doi:10.3389/fmicb.2025.1619546)

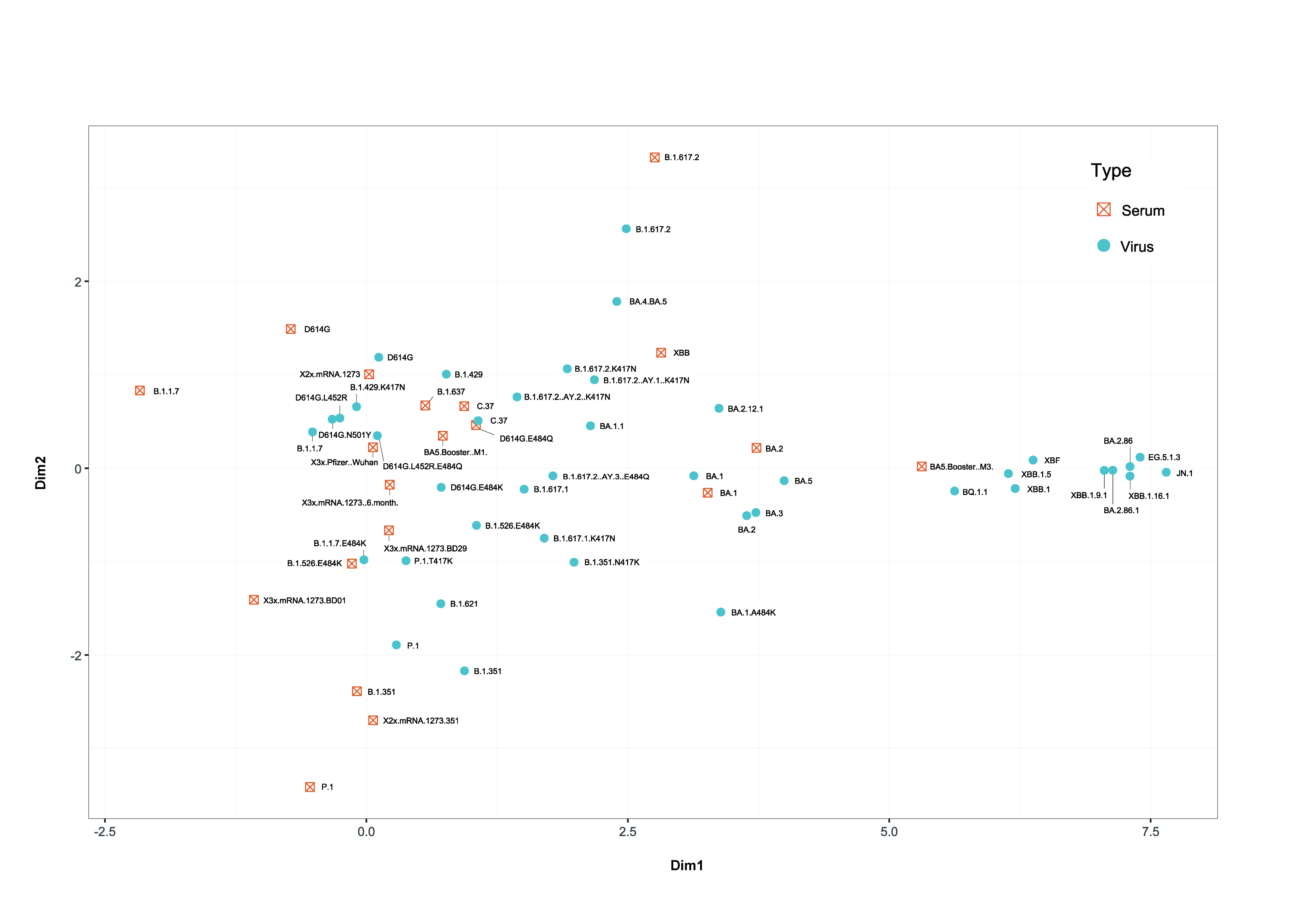

Supplement: Supplementary file 2 [file Data_Sheet_2.zip › S1 fig.tif]

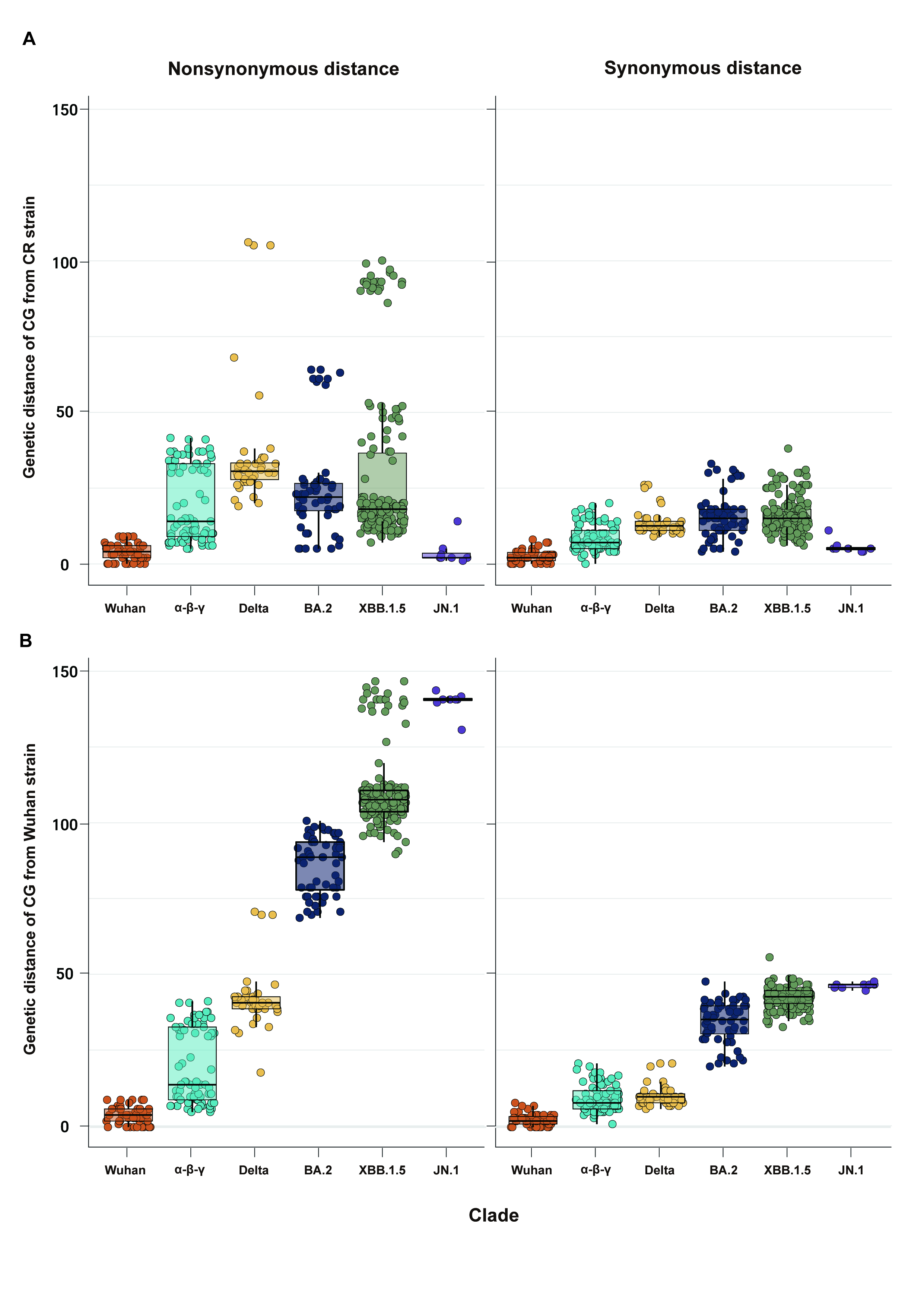

Supplement: Supplementary file 2 [file Data_Sheet_2.zip › S2 fig.tif]

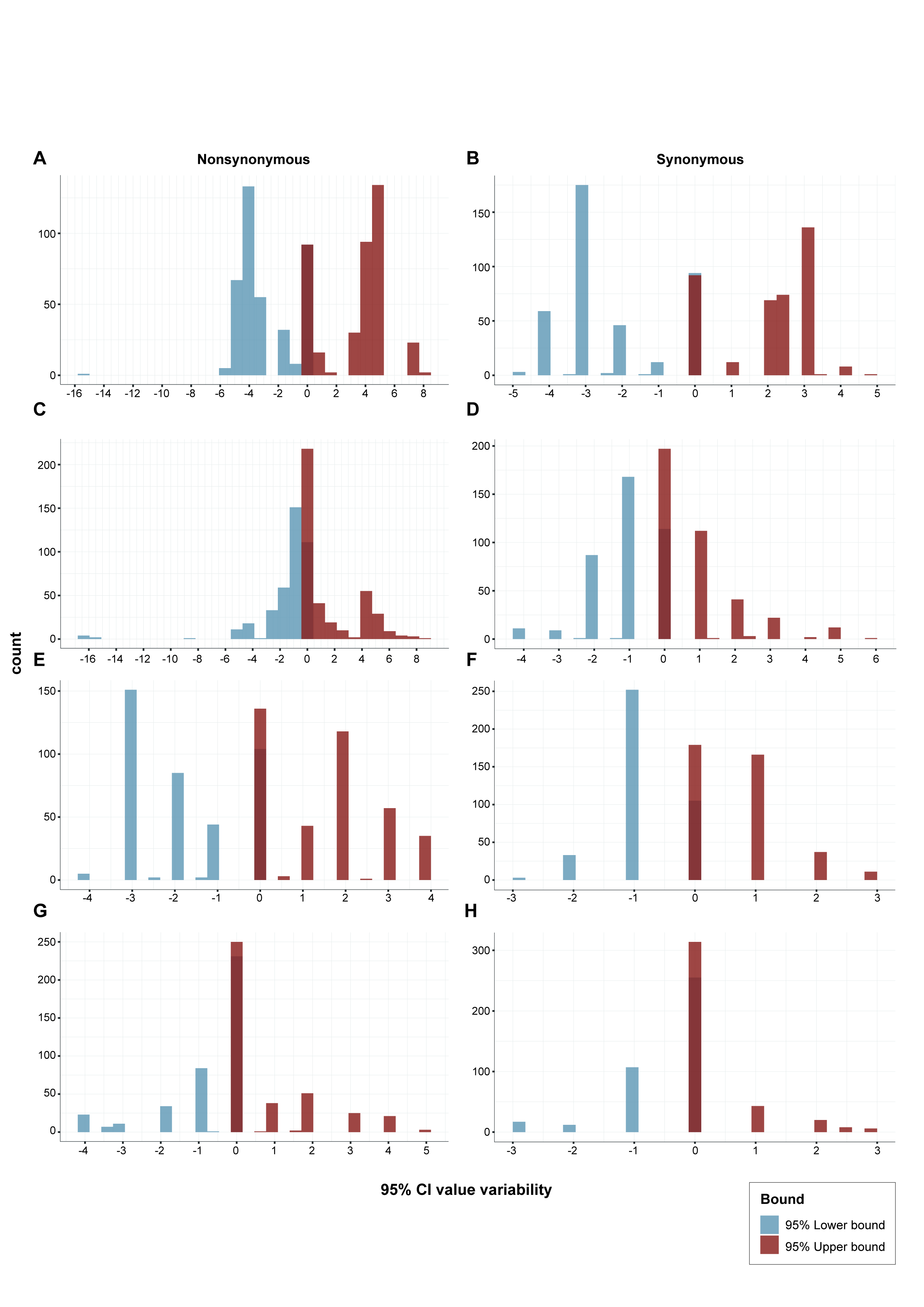

Supplement: Supplementary file 2 [file Data_Sheet_2.zip › S3 fig.tif]

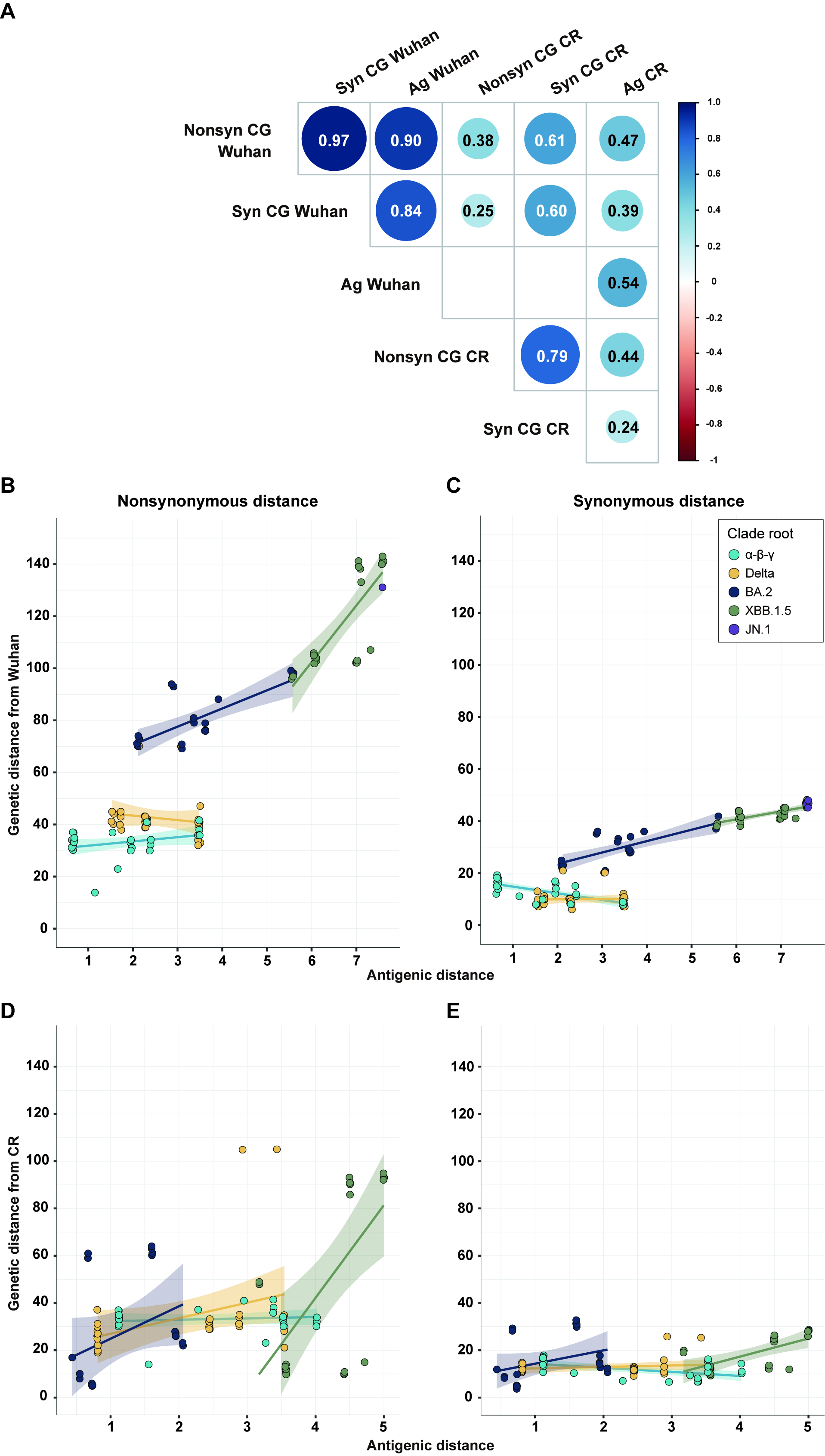

Supplement: Supplementary file 2 [file Data_Sheet_2.zip › S4 fig.tif]

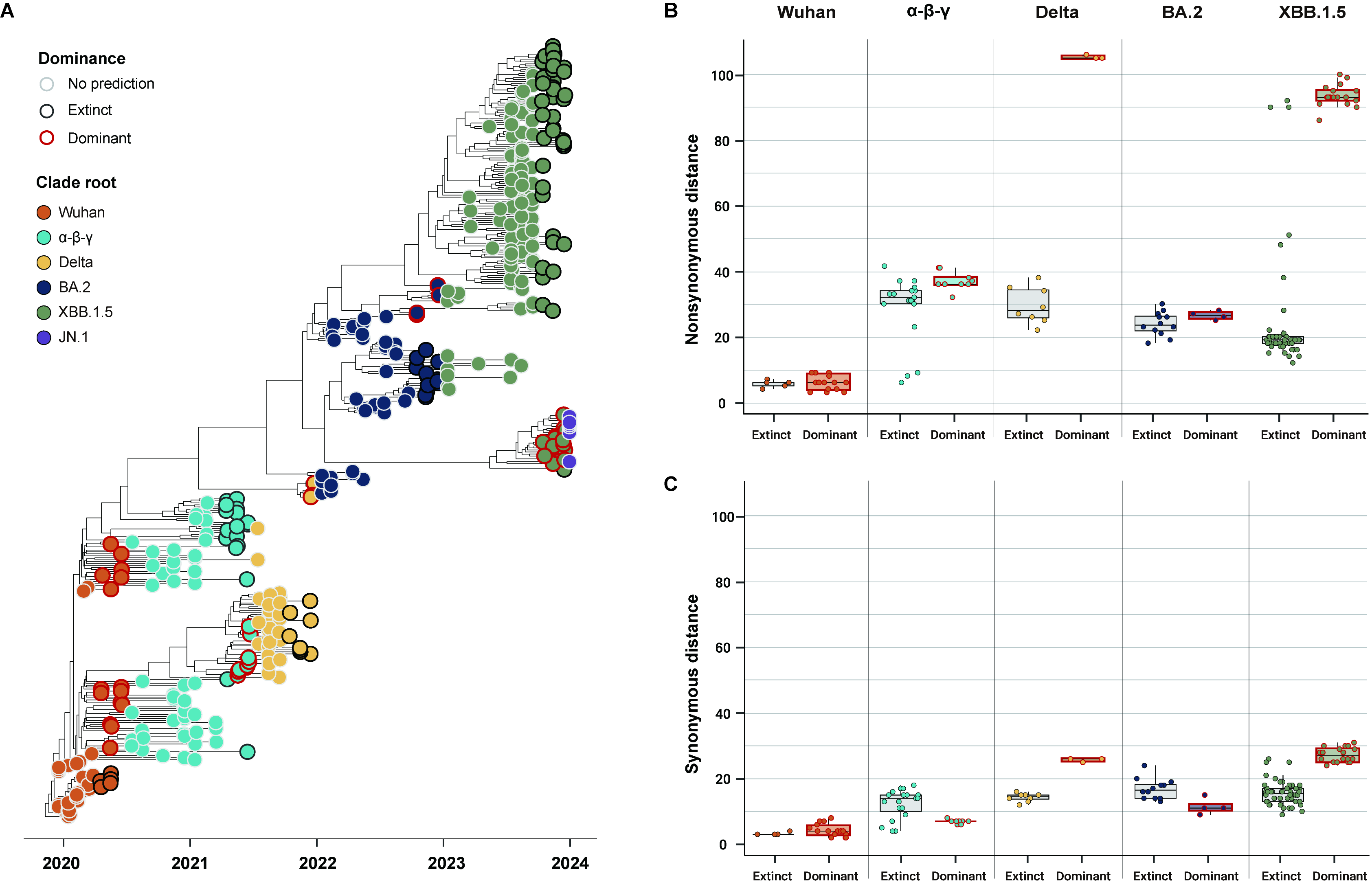

Supplement: Supplementary file 2 [file Data_Sheet_2.zip › S5 fig.tif]

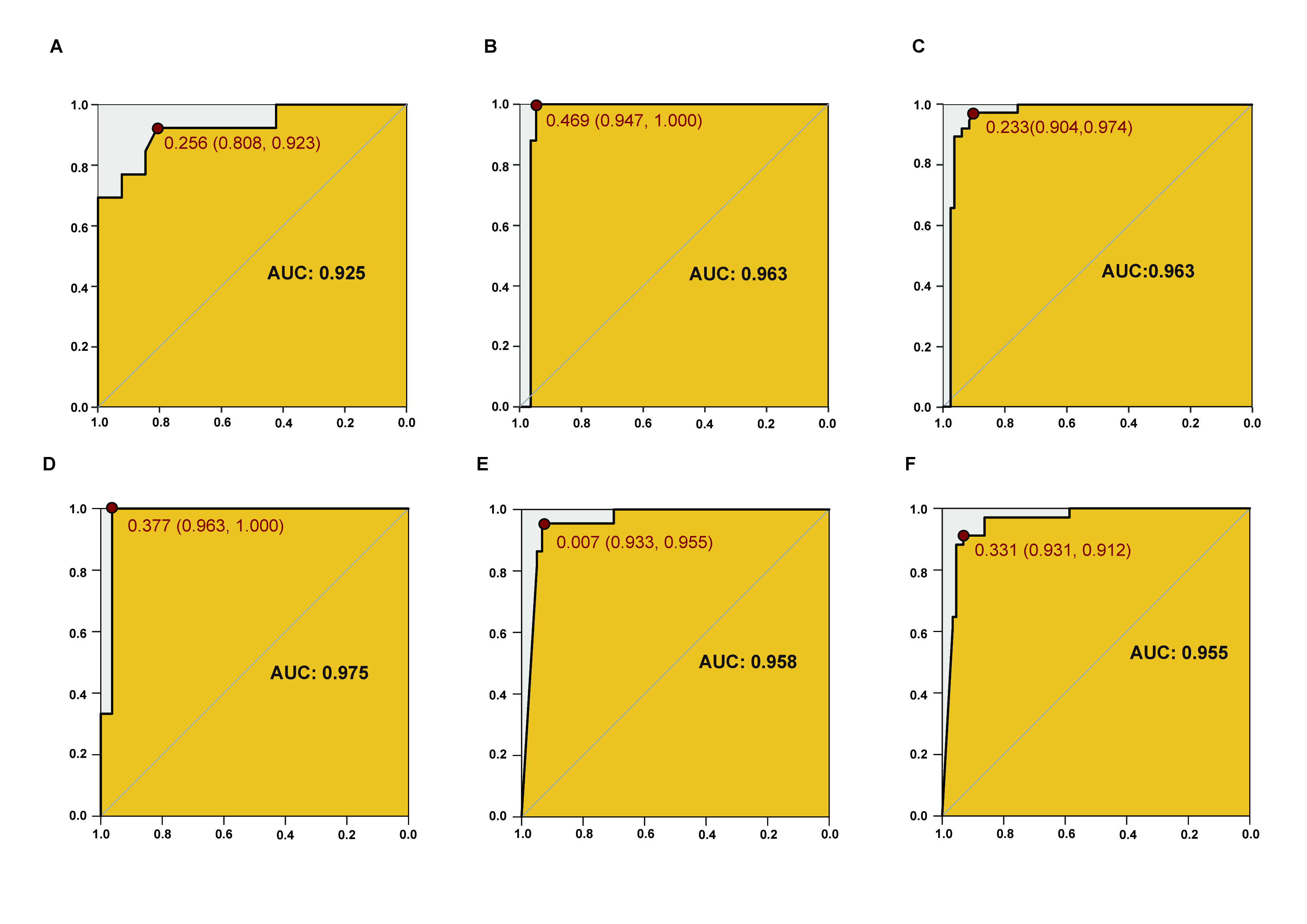

Supplement: Supplementary file 2 [file Data_Sheet_2.zip › S6 fig.tif]

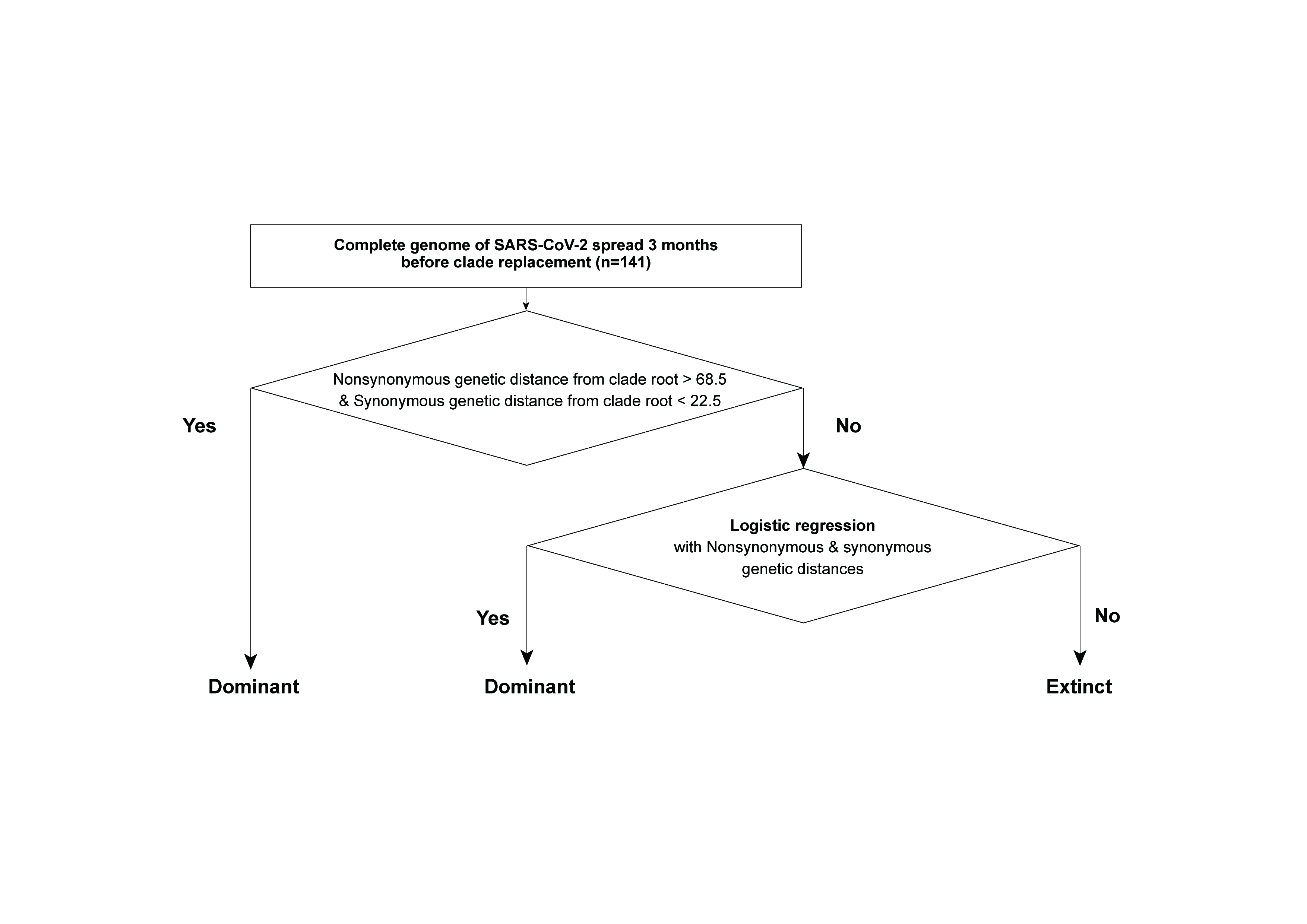

Supplement: Supplementary file 2 [file Data_Sheet_2.zip › S7 fig.tif]
